# Incidence of infant mortality and its predictors in East Africa using Gompertz gamma shared frailty model

**DOI:** 10.1186/s13690-022-00955-7

**Published:** 2022-08-23

**Authors:** Getayeneh Antehunegn Tesema, Beminate Lemma Seifu, Zemenu Tadesse Tessema, Misganaw Gebrie Worku, Achamyeleh Birhanu Teshale

**Affiliations:** 1 Department of Epidemiology and Biostatistics, Institute of Public Health, College of Medicine and Health Science, University of Gondar, Gondar, Ethiopia; 2 Department of Public Health, College of Medicine and Health Sciences, Samara University, Samara, Ethiopia; 3 Department of Human Anatomy, School of Medicine, School of Medicine and Health Sciences, University of Gondar, Gondar, Ethiopia

**Keywords:** Infant mortality, East Africa, Gompertez gamma shared frailty modeling

## Abstract

**Background:**

Globally, infant mortality is a major public health concern and a sensitive indicator of countries' socio-economic and health status. Despite the substantial reduction of under-five mortality in sub-Saharan African countries specifically in East Africa, the infant mortality rate remains highest and too far below to achieve the WHO target. As to our search of the literature is concerned, there is a dearth of evidence on the incidence and predictors of infant mortality in East Africa. Therefore, this study investigated the incidence of infant mortality and its predictors in East Africa.

**Methods:**

The present study has utilized 138,803 weighted samples from Demographic and Health Surveys (DHSs) of 12 East African countries. Considering the hierarchical nature of DHS data shared frailty parametric survival models were fitted and compared based on deviance (-2LLR), AIC, and BIC. Gompertz gamma shared frailty model was the best-fitted model for the data since it had the lowest deviance, AIC, and BIC values. Variables with a *p*-value < 0.2 in the bi-variable analysis were considered for the multivariable analysis. In the multivariable Gompertz gamma shared analysis, the Adjusted Hazard Ratio (AHR) with 95% Confidence Interval (CI) was reported to declare the significant predictors of infant mortality.

**Results:**

The infant mortality rate in East Africa was 41.41 per 1000 live births. Mothers aged 25–34 years, wanted birth, health facility delivery, 1–3 ANC visit, being 2^nd^- 4^th^ birth order, 5^th^ and above, the birth interval of 24–48 months, and birth interval of 49 months and above were significantly associated with lower risk of infant mortality. Whereas women who didn’t have formal education, women who didn't participate in making health care decisions making, being male children, cesarean delivery, small size at birth, and large size at birth were significantly associated with a higher risk of infant mortality.

**Conclusion:**

Despite the substantial progress in improving maternal and child health, this study showed that infant mortality is still a major public health concern in East Africa. Maternal age, place of delivery, maternal education, birth size, sex of the child, mode of delivery, women's autonomy, birth order, birth interval, and ANC visit were found to be significant predictors of infant mortality. Therefore, public health interventions enhancing health facility delivery, ANC visit, maternal education, birth spacing, and empowering women are crucial for reducing the incidence of infant mortality in East Africa.

## Background

Despite the remarkable reduction in infant mortality worldwide, 4.1 million children still died every year before their first birthday, accounting for 73% of under-5 mortality [1]. The global infant mortality rate decreased from 65 deaths per 1000 live births in 1990 to 29 deaths per 1000 live births in 2017 [2]. Infant mortality is one of the most important indicators of population health and has important implications for countries' economic development, social well-being, and access to medical care [3, 4].

Notwithstanding the substantial reduction in under-five mortality, infant mortality remains high and continues as a major public health challenge [1, 5–7]. The deaths are primarily concentrated in Sub-Saharan Africa specifically East Africa, which has the greatest infant mortality rate (92.2% per 1000 live births) when compared to developed countries (8 death per 1000 birth) [2, 8]. It is primarily caused by preventable factors that can be eradicated by providing necessities, and it is seen as a reflection of disparities in infrastructure, services, and socioeconomic development [9]. Almost 80% of infant deaths could have been avoided, and the majority (51.3%) could have been reduced if mothers had received proper care during their pregnancy and infants had received basic care [10].

Infant mortality is still a public health issue around the world, particularly in low-income countries [11], and is used as an important woman and child health indicator [12, 13]. However, despite progress made by many countries to achieve the Millennium Development Goals (MDG) 4 to reduce child mortality by two-thirds between the years 1990 and 2015 [14], half of the world’s nations are still behind their targets [15]. Millions of babies are estimated to die in the first year of life in East Africa but most die at home, remain uncounted, and are invisible to public health programs[16, 17]. Unlike developed countries, East Africa's reduction in infant mortality has shown slow progress, due to the continued huge burden of pneumonia, diarrhea, malaria, and vaccine-preventable diseases [18–20]. Previous literature documented that infant mortality was significantly associated with place of delivery [21], mode of delivery [22], Antenatal Care (ANC) visit during pregnancy [23], preceding birth interval [24], maternal education [25–27], media exposure [28], and place of residence [21, 29]. Besides, women's health care decision-making [30–32], household wealth status [33–35], distance to a health facility [12, 36], birth order [37], wanted pregnancy [38], childbirth size [39], multiple births [40] and sex of child [41] were found to be significant predictors of infant mortality.

Despite East African countries sharing the huge burden of global infant mortality, as to our search of the literature, there is limited evidence on the pooled incidence and predictors of infant mortality in East Africa. Therefore, this study aimed to investigate the predictors of infant mortality in East Africa based on the pooled DHS data in 12 East African countries. Given DHS data nature, studying the effect of community clustering on infant mortality has both research and policy implications. We can determine the risk factors of infant mortality, accounting for the correlated observations of live births in the same cluster. Having identified the literature gaps to capture the incidence and predictors of infant mortality, the present study investigated whether there are dependencies between study subjects within the same community, that is, to check if the children share similar frailty within the community.

## Methods and materials

### Study design and area

The Demographic and Health Surveys (DHSs) of the 12 East African countries were the data source for the present study. These were Burundi, Ethiopia, Comoros, Uganda, Rwanda, Tanzania, Mozambique, Madagascar, Zimbabwe, Kenya, Zambia, and Malawi. The DHS is a nationally representative survey that provides data for monitoring indicators of population dynamics, nutrition, and health. The permission to use the data was granted from the measure DHS program.

### Study population and sampling

Newborns from birth to the first year of birth in 12 East African countries were the source of the population whereas those found in the selected Enumeration Areas (EAs) or clusters were the study population. A multi-stage cluster sampling technique was employed to recruit the samples using EAs and households as primary and secondary sampling units, respectively. In DHS, some of the regions or counties were oversampled and some were undersampled specifically in large counties or regions. Therefore, we have applied sample weighting for the computation of means, percentages, and regression as per the DHS recommendation to restore the representativeness of the data at the national and sub-national levels. for this study. The most recent births of the mother were considered for this study and the data were extracted from the Kids Record (KR) file. A total of 138,803 most recent live births were considered for analysis.

### Study variables

#### Dependent variable

The time to death of the children before their first birthday was the dependent variable. Children who died in the first year of life were considered as having the event and coded as 1 whereas those who were not were considered censored and coded as 0. The information about child survival was obtained retrospectively by interviewing the mother. Age at death was recorded in months.

#### Explanatory variables

The explanatory variables were categorized into three themes. 1) Demographic variables; residence, marital status, maternal age, and country, 2) socio-economic variables; maternal education, husband education, wealth status, media exposure, and maternal occupation, and 3) maternal obstetric and child-related variables were child age, sex of the child, birth order, birth size, birth outcome, birth size, place of delivery, mode of delivery, women's health care decision making autonomy, unwanted pregnancy, number of ANC visits, preceding birth interval and distance to the health facility.

### Statistical analysis

All the reported results were based on the weighted data and STATA version 17 and R version 3.5.1 software were used for analysis. The global Schoenfeld residuals test (both global and scaled) and graphical methods were used to check the proportional hazard assumption. When the *p*-value < 0.05, indicated the proportional hazard assumption was violated, this indicates the Cox-proportional model is not an appropriate model. Because the proportional hazard assumption was violated, the Cox-proportional model was ruled out. Unlike the Cox model, the parametric survival models assume a particular distribution whose parameters depend on the covariates. We compared the equality of the survival curve across population groups using a log-rank test with the null hypothesis of no difference between two or more survival distributions at any point in time.

Given the hierarchical nature of the DHS data, infants were nested within EAs. There might be the possibility of a clustering effect and therefore the fundamental assumption of the classical regression model i.e. equal variance and independence of observations. Parametric survival models were fitted. Then we checked whether there is clustering or not by fitting the frailty model (random effect survival model) and the theta value was significant in the null model, if its *p*-value is < 0.05, indicates that there is unobserved heterogeneity or shared frailty. Therefore, infants in one cluster were more related to each other than infants in other clusters. In addition, the LR-test assesses whether the shared frailty model was the best-fitted model compared to the classical model for the data. Frailty can't be directly estimated from the data, it is assumed to follow a distribution with mean = 1 and variance = 0. If the frailty is less than 1, the subjects are less likely to be frail, and if greater than 1, the subjects are more likely to be frail. In this study, frailty was modeled according to the number of EAs. Besides, the EDHS data structure has hierarchical nature and we have checked whether there is clustering or not by fitting the frailty model (random effect survival model) and the theta was significant in the null model (θ = 0.03, 95% CI: 0.01, 0.05), LR test of theta = 0: chibar2 (01) = 12.35, Prob >  = chibar2 < 0.001). It suggests that there is unobserved heterogeneity or shared frailty. As a result, infants in one cluster were more closely related than those in other groups. Furthermore, because the LR-test was significant, the shared frailty model was the best-fitted model for the data. The Gompertz gamma shared frailty model was shown to be the best-fitting of five parametric models.

Nested parametric models in generalized gamma (Exponential, Weibull, lognormal) were compared based on deviance, and non-nested models (Gompertz and log-logistic) were compared using AIC. Deviance, AIC, and Cox-Snell residual graph. Based on the above-mentioned comparison parameters, the Gompertz gamma shared frailty model was found the best-fitted model for the data.

Variables with a p-value less than 0.20 in the uni-variable gamma shared frailty analysis were included in the multivariable analysis. We estimate the hazard ratio and 95% confidence interval. In the multivariable analysis, the Adjusted Hazard Ratio (AHR) with 95% Confidence Interval (CI) was used to declare significant predictors of infant mortality.

### Ethical consideration

Permission for data access was obtained from the measure DHS program through an online request from http://www.dhsprogram.com. The data used for this study were publicly available with no personal identifier.

## Results

### Descriptive results of the study participants

A total of 138,803 most recent live births were included. About 66,070 (47.6%) of the children were born to mothers aged 25–34 years and more than three-quarters of the respondents (78.3%) were rural residents. About 33,229 (23.9%) and 23,298 (16.8%) of the children belonged to the poorest and richest households, respectively. Nearly one-fourth (24.1%) of the children were born to mothers who didn't have formal education, and the majority (64.9%) of the respondent had no media exposure. Regarding birth size, about 64,315 (46.3%) of them were average size at birth (Table 1).

**Table 1:** Descriptive characteristics of the study participants

Variable	Weighted frequency	Percentage (%)
**Country**		
Burundi	13611	9.8
Ethiopia	11022	7.9
Kenya	19563	14.1
Comoros	3235	2.3
Madagascar	12686	9.1
Malawi	17395	12.5
Mozambique	11704	8.4
Rwanda	8003	5.8
Tanzania	10052	7.2
Uganda	15270	11.0
Zambia	9841	7.1
Zimbabwe	6418	4.6
**Residence**		
Rural	30108	21.7
Urban	108695	78.3
**Maternal age**		
15–24	41683	30.0
25–34	66070	47.6
≥ 35	31050	22.4
**Maternal education status**		
No	33448	24.1
Primary	73811	53.2
Secondary and above	31544	22.7
**Husband education status**		
No	22654	16.3
Primary	57352	41.3
Secondary and above	58797	42.4
**Wealth status**		
Poorest	33229	23.9
Poorer	29866	21.5
Middle	26820	19.3
Richer	25590	18.4
Richest	23298	16.8
**Media exposure**		
No	48776	35.1
Yes	90027	64.9
**Marital status**		
Single	6482	4.7
Married	118613	85.4
Divorced/widowed/separated	13708	9.9
**Respondent working**		
No	44616	32.1
Yes	94187	67.9
**Sex of child**		
Male	70206	50.6
Female	68597	49.4
**Birth outcome**		
Single	134388	96.8
Multiple	4415	3.2
**Birth size**		
Large	40812	29.4
Average	64315	46.3
Small	33676	24.3
**Birth order**		
First	32654	23.5
2–4	66883	48.2
≥ 5	39266	28.3
**Place of delivery**		
Home	38005	27.4
Health facility	100798	72.6
**Mode of delivery**		
Vaginal	130518	94.0
Cesarean delivery	8285	6.0
**Women’s health care decision making autonomy**		
Respondent alone	84970	18.6
Jointly with their husband/parent	55653	40.1
Husband/parent alone	57402	41.3
**Distance to a health facility**		
A big problem	84970	61.2
Not a big problem	53833	38.8
**Unwanted pregnancy**		
No	82691	59.6
Yes	56112	40.4
**Number of ANC visit**		
No	6127	4.4
1–3	39791	28.7
≥ 4	92885	66.9
**Preceding birth interval (in months)**		
< 24	18963	13.7
24–48	58591	42.2
≥ 49	61249	44.1
**Weight/age**		
Normal	126731	91.3
Moderately underweight	9089	6.6
Severely underweight	2983	2.2
**Height/age**		
Normal	107654	77.6
Moderately stunted	19032	13.7
Severely stunted	12117	8.7
**Weight/height**		
Normal	134523	96.9
Moderately wasted	3044	2.2
Severely wasted	1236	0.9

### Infant mortality rate

**Fig. 1:**
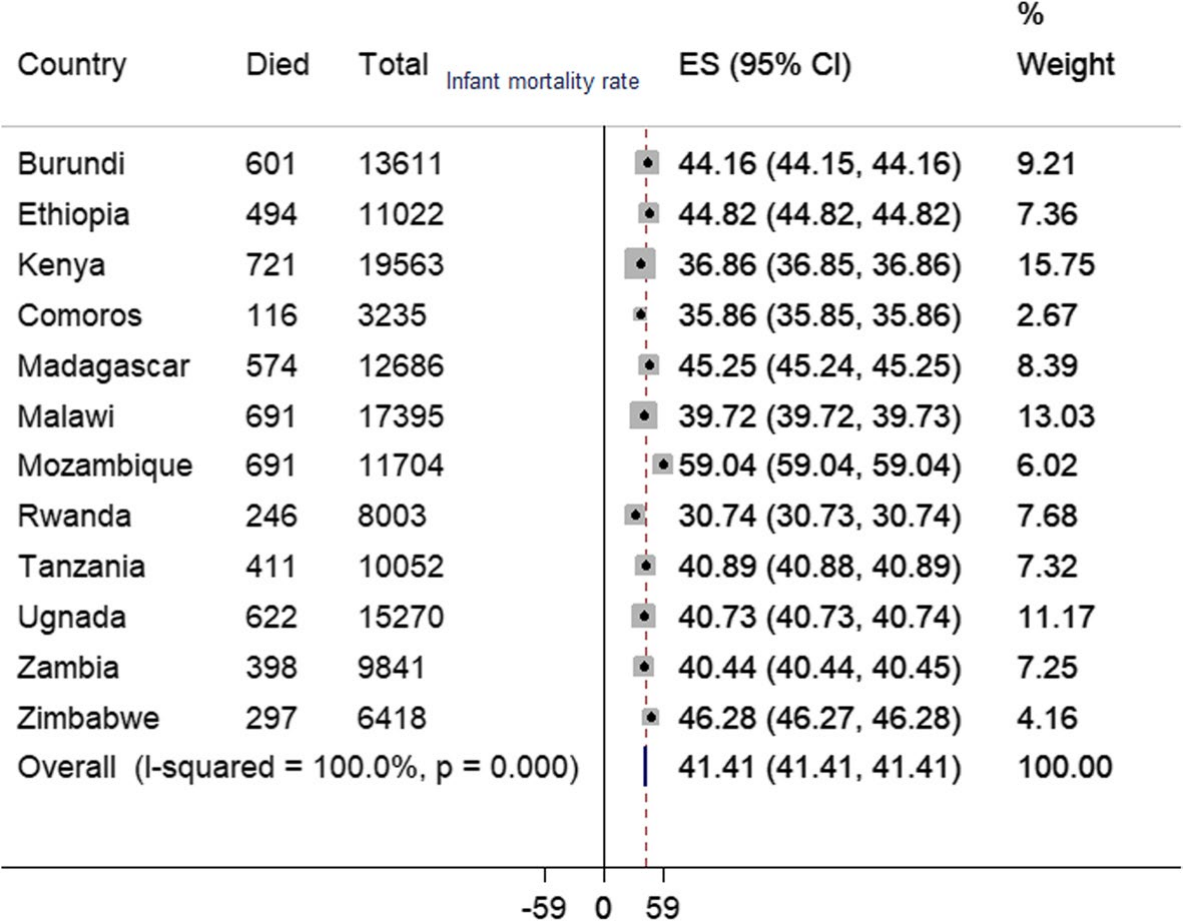
The infant mortality rate in East African countries

From the total of 138,803 most recent live births, the overall infant mortality in East Africa was 41.41 (95% CI: 41.41, 41.1) per 1000 live births, and it was varied across regions (Fig. 1).

### Comparison of failure functions

**Fig. 2:**
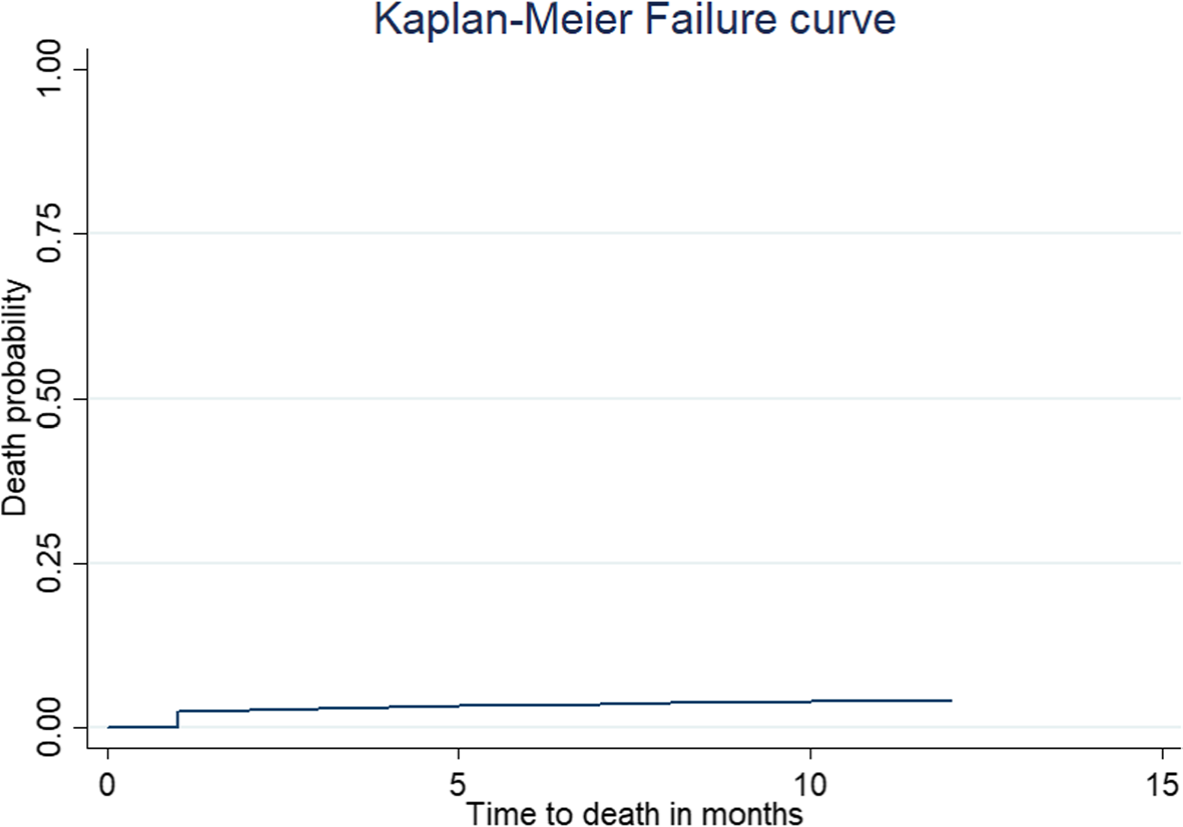
The Kaplan–Meier failure curve of infant mortality in East Africa

The Kaplan–Meier failure curve was used to compare the probability of mortality across categorical explanatory variables visually and log-rank test objectively. The overall Kaplan–Meier failure curve indicated that the probability of infant mortality increased over time (Fig. 2). There was a statistically significant difference in infant mortality across the residence, country, mode of delivery, birth order, ANC, birth outcome, place of delivery, preceding birth interval, health insurance coverage, distance to the health facility, wanted pregnancy, birth size, maternal education, husband education, respondent age, twin pregnancy, and wealth index (log-rank, *p* < 0.05) (Table 2).

**Table 2:** Log-rank test for the predictors of infant mortality

Variable	*P*-value	Variable	*P*-value
Residence	0.018	Multiple births	< 0.001
Wealth index	0.0003	Maternal age	< 0.001
Sex of child	< 0.0001	Media exposure	0.67
Country	< 0.0001	Wanted pregnancy	< 0.0001
Maternal education	< 0.0001	Distance to a health facility	0.0002
Husband education	0.04	Covered by health insurance	< 0.001
ANC visit	< 0.0001	Women’s decision making autonomy	0.014
Preceding birth interval	< 0.0001	Birth order	< 0.0001
Mode of delivery	0.0002	Birth size	< 0.001
Place of delivery	0.003		

### Assessing proportional hazard assumption

Proportional hazard assumption was checked using the global Schoenfeld residuals test (global and scaled) for all possible predictors of infant mortality. The global Schoenfeld residual test and the *p*-value was < 0.05 (Table 3).

**Table 3:** Schoenfeld residual test for checking proportional hazard assumption for the incidence of infant mortality and its predictors among live births in the 12 East African countries

Variables	Rho	Chi2	Df	Prob > chi2
Residence	0.006	0.23	1	0.62
Country	-0.022	3.54	1	0.06
Wealth index	-0.04	12.48	1	0.0004
Sex of child	0.045	16.53	1	< 0.001
Women education	-0.02	2.95	1	0.09
Husband education	-0.034	8.53	1	0.004
Place of delivery	-0.022	3.56	1	0.06
ANC visit	0.084	58.48	1	< 0.001
Birth interval	-0.006	0.23	1	0.63
Mode of delivery	-0.03	5.54	1	0.02
Birth outcome	-0.08	42.99	1	< 0.001
Maternal age	0.04	13.76	1	0.0002
Media exposure	-0.011	0.90	1	0.34
Wanted pregnancy	0.018	2.24	1	0.13
Distance to a health facility	-0.017	2.04	1	0.15
Covered by health insurance	0.0006	0.001	1	0.96
Women autonomy	0.0007	0.001	1	0.95
Maternal age	0.003	0.07	1	0.79
Birth order	-0.0019	0.03	1	0.987
Birth size	-0.093	71.41	1	< 0.001
Global test		333.84	20	< 0.0001

### Predictors of infant mortality

In the multivariable Gompertz gamma shared frailty model; country, maternal age, maternal education status, wanted pregnancy, women health care decision-making autonomy, sex of a child, place of delivery, number of ANC visits, mode of delivery, twin birth, birth order, birth size and preceding birth interval were significant predictors of infant mortality. Child in Burundi, Madagascar, Malawi, Mozambique, Tanzania, Uganda, Zambia and Zimbabwe were 1.34 (AHR = 1.34, 95% CI: 1.15, 1.56), 1.20 (AHR = 1.20, 95% CI: 1.03, 1.41), 1.29 (AHR = 1.29, 95% CI: 1.11, 1.50), 1.68 (AHR = 1.68, 95% CI: 1.44, 1.96), 1.23 (AHR = 1.23, 95% CI: 1.04, 1.44), 1.18 (AHR = 1.18, 95% CI: 1.01, 1.37), 1.29 (AHR = 1.29, 95% CI: 1.09, 1.52) and 1.45 (AHR = 1.45, 95% CI: 1.21, 1.74) times higher hazard of death in the first year of life compared to child born in Rwanda respectively. The hazard of infant mortality among children born to mothers aged 25–34 years was decreased by 11% (AHR = 0.89, 95% CI: 0.83, 0.96) than a child born to a mother aged 15–24 years. Mothers who didn’t attend formal education had 1.23 times (AHR = 1.23, 95% CI: 1.13, 1.33) higher hazard of infant mortality than mothers who attained secondary education and higher. Wanted births had decreased the hazard of infant mortality by 32% (AHR = 0.68, 95% CI: 0.64, 0.72) compared to unwanted births. Births born to mothers who didn't participate in making their own health care decision were 1.12 times (AHR = 1.12, 95% CI: 1.04, 1.21) higher hazard of infant mortality compared to a child born to a mother who made their own health care decision. The hazard of infant mortality among male children was increased by 33% (AHR = 1.33, 95% CI: 1.27, 1.43) than female children. The hazard of infant mortality among health facility delivered and having 1–3 ANC visits were decreased by 10% (AHR = 0.90, 95% CI: 0.84, 0.97) and 41% (AHR = 0.59, 95% CI: 0.51, 0.67) than who delivered at home and who didn't have ANC visit during pregnancy, respectively. Cesarean deliveries were 1.13 times (AHR = 1.13, 95% CI: 1.01, 1.25) higher than vaginal deliveries. Multiple births had 4.18 times (AHR = 4.18, 95% CI: 3.84, 4.55) higher hazard of infant mortality than singletons. The hazard of infant mortality among children who were second or fourth birth and above four was lowered by 39% (AHR = 0.61, 95% CI: 0.55, 0.67), and 29% (AHR = 0.71, 95% CI: 0.63, 0.80) compared to first-order birth, respectively. Children who were small and large size at birth were 1.87 times (AHR = 1.87, 95% CI: 1.75, 1.99) and 1.08 times (AHR = 1.08, 95% CI: 1.01, 1.15) at higher hazard of infant mortality than average size child at birth respectively. The hazard of infant mortality among children born to mothers who had a preceding birth interval of 24–48 months and ≥ 49 months were decreased by 49% (SHR = 0.51, 95% CI: 0.47, 0.55) and 51% (AHR = 0.49, 95% CI: 0.45, 0.54) than a child born to mother who had less than 24 months, respectively (Table 4).

**Table 4:** Bi-variable and multivariable Gompertz gamma shared frailty analysis of predictors of infant mortality in East African countries

Variables	Infant mortality		CHR with 95% CI	AHR with 95% CI
	**Alive**	**Died**		
**Residence**				
Urban	28829	1279	1	1
Rural	104107	4588	1.08 (1.01, 1.15)	0.93 (0.88, 1.03)
**Country**				
Rwanda	7754	249	1	1
Burundi	13010	601	1.16 (1.16, 1.57)	1.34 (1.15, 1.56)*
Ethiopia	10528	495	1.55 (1.33, 1.80)	1.15 (0.98, 1.36)
Kenya	18842	721	1.13 (0.98, 1.31)	0.89 (0.76, 1.04)
Comoros	3119	116	1.11 (0.89, 1.39)	0.85 (0.68, 1.08)
Madagascar	12,112	574	1.48 (1.27, 1.72)	1.20 (1.03, 1.41)*
Malawi	16,704	691	1.28 (1.11, 1,48)	1.29 (1.11, 1.50)*
Mozambique	11,013	691	1.92 (1.66, 2.22)	1.68 (1.44, 1.96)*
Tanzania	9641	411	1.31 (1.12, 1.53)	1.23 (1.04, 1.44)*
Uganda	14648	622	1.34 (1.16, 1.55)	1.18 (1.01, 1.37)*
Zambia	9442	398	1.31 (1.12, 1.54)	1.29 (1.09, 1.52)*
Zimbabwe	6121	297	1.41 (1.19, 1.68)	1.45 (1.21, 1.74)*
**Maternal age (in years)**				
15–24	39739	1943	1	1
25–34	63530	2540	0.80 (0.75, 0.84)	0.89 (0.83, 0.96)*
≥ 35	29667	1383	0.94 (0.88, 1.01)	1.05 (0.95, 1.16)
**Maternal education status**				
No	31972	1476	1.24 (1.14, 1.34)	1.23 (1.13, 1.33)*
Primary	70614	3197	1.22 (1.14, 1.31)	1.10 (0.99, 1.22)
Secondary and above	30350	1194	1	1
**Husband education status**				
No	21694	961	1.09 (1.01, 1.17)	0.95 (0.87, 1.04)
Primary	54853	2499	1.06 (0.99, 1.12)	1.01 (0.95, 1.08)
Secondary and above	56389	2408	1	1
**Wealth status**				
Poorest	31763	1465	1.20 (1.11, 1.31)	1.08 (0.96, 1.20)
Poorer	28584	1282	1.15 (1.06, 1.26)	1.09 (0.97, 1.21)
Middle	25712	1108	1.09 (0.99, 1.19)	1.04 (0.94, 1.16)
Richer	24473	1118	1.12 (1.02, 1.22)	1.09 (0.98, 1.20)
Richest	22403	894	1	1
**Wanted pregnancy**				
No	78794	3897	1	1
Yes	54141	1970	0.73 (0.69, 0.78)	0.68 (0.64, 0.72)**
**Distance to a health facility**				
Not a big problem	81481	3489	1	1
A big problem	51455	2378	1.10 (1.05, 1.17)	1.02 (0.96, 1.08)
**Women’s health care decision making autonomy**				
Respondent alone	24711	1037	1	1
Jointly with their husband/parent	53323	2330	1.06 (0.98, 1.14)	1.04 (0.96, 1.12)
Husband/parent alone	54901	2501	1.11 (1.03, 1.20)	1.12 (1.04, 1.21)*
**Sex of child**				
Male	66882	3324	1.29 (1.22, 1.36)	1.33 (1.27, 1.43)*
Female	66053	2543	1	1
**Place of delivery**				
Home	36337	1668	1	1
Health facility	96599	4199	0.92 (0.87, 0.97)	0.90 (0.84, 0.97)**
**Number of ANC visit**				
No	5788	339	1	1
1–3	38644	1146	0.51 (0.45, 0.58)	0.59 (0.51, 0.67)*
≥ 4	88503	4382	0.85 (0.76, 0.95)	0.92 (0.81, 1.03)
**Mode of delivery**				
Vaginal	125078	5439	1	1
Cesarean delivery	7857	428	1.22 (1.10, 1.35)	1.13 (1.01, 1.25)*
**Twin birth**				
No	129265	5123	1	1
Yes	3671	744	4.62 (4.27, 5.01)	4.18 (3.84, 4.55)**
**Birth order**				
1	31065	1588	1	1
2–4	64408	2475	0.74 (0.69, 0.79)	0.61 (0.55, 0.67)*
≥ 5	37462	1804	0.94 (0.88, 1.06)	0.71 (0.63, 0.80)*
**Birth size**				
Average	39261	1551	1	1
Small	62038	2277	1.81 (1.70, 1.92)	1.87 (1.75, 1.99)**
Large	31636	2039	1.08 (1.01, 1.15)	1.08 (1.01, 1.15)**
**Preceding birth interval (in months)**				
< 24	17776	1187	1	1
24 – 48	56550	2041	0.54 (0.50, 0.58)	0.51 (0.47, 0.55)**
≥ 49	58609	2640	0.67 (0.62, 0.72)	0.45, 0.54)*

*CHR* Crude Hazard Ratio, *CI* Confidence Interval, *AHR* Adjusted Hazard Ratio

^*^P-value < 0.05

^**^P-value < 0.001*,*

## Discussion

The present study revealed infant mortality rate in East Africa was 41.41 (95% CI: 41.41, 41.1) per 1000 live births. It was higher than the WHO target [42], it might be due to sub-Saharan African countries specifically East Africa continuing to be the host spot areas of infectious disease, malnutrition, and poor health care access, which in turn directly or indirectly responsible for infant mortality and morbidity [43]. Besides, East Africa faces extreme poverty, poor education, low health knowledge, poor infrastructure, lifestyle, and environmental factors (i.e., limited access to resources such as clean water) that have been identified as primary factors contributing to the high incidence of infant mortality [44, 45].

Maternal age, maternal education status, wanted pregnancy, women health care decision-making autonomy, sex of a child, place of delivery, number of ANC visits, mode of delivery, twin birth, birth order, birth size, and preceding birth interval found to be significant predictors of infant mortality. Maternal education and participation in making health care decisions were found significantly associated with a lower risk of infant mortality. It was in line with studies in Nicaragua [25], Denmark [46], Bangladesh [47], and Pakistan [48]. The possible explanation might be due to maternal education plays a significant role in adopting healthy behaviors and habits that have a positive impact on their child's health and more capable of getting quality care for their children [49]. Women participating in making health care decision-making can make key decisions about their own and their children, such as compliance with vaccination schedules, provision of recommended nutrition, and having a good awareness of childhood illness, these could be responsible for the reduced risk of infant mortality among children born to mothers who have decision making autonomy [50]. In line with study findings reported in Low and middle-income countries (LMIC) [51] and South Asia [52], children born to mothers aged 25–34 years had a lower hazard of infant mortality than children born to mothers aged 15–24 years. The higher risk of infant mortality among births to adolescent mothers could be due to nutritional insufficiencies, because of mother-fetus and infants’ competition for nutrients, as adolescents still require additional energy for growth and development [26]. In addition, teenage pregnancy is a high-risk pregnancy and increases the risk of obstetric complications and adverse child outcomes [53–57]. moreover, studies affirmed that teenagers are less likely to use maternal health care services such as ANC, institutional delivery, PNC, and routine immunization which could increase the risk of infant mortality [58].

Being male increases the hazard of infant mortality to female children. This was supported by a study reported in Pakistan [48], this can be due to males being more vulnerable to morbidities such as low Apgar score, Intra-uterine Growth Restriction (IUGR), respiratory insufficiency, and prematurity than the female sex [59]. Besides, it might be because of a higher level of testosterone among males and this could affect pulmonary biomechanics and vascular development that could make males more vulnerable to respiratory and neurological diseases [60, 61]. In line with studies reported in Bangladesh [62], SSA [63], and India [48], health facility delivery and ANC visits were significantly correlated with decreased risk of infant mortality. It could be due to the reason that ANC visit is an entry point for the other maternal health services, and births from mothers who had no ANC visit are not aware of danger signs of pregnancy and underlying medical conditions that could lead to low birth weight, prematurity, congenital anomalies as compared to women who had ANC visit [64]. Cesarean delivery was significantly associated with an increased risk of infant mortality. It was consistent with studies reported in Nepal [65] and industrialized countries [66]. Though cesarean delivery is life-saving for the mother and the baby during emergency conditions, they are at risk of developing complications due to surgical procedures. Compared to babies born vaginally, babies born by cesarean are at risk for health complications they are more likely to have difficulty breathing on their own and are born preterm before the lungs have fully developed. Mothers who gave birth through cesarean section, mothers and babies are less likely to have skin-to-skin contact immediately after birth, and because of the drug for anesthesia the mother sedates make them not initiate breastfeeding immediately, and the newborn's mouth, esophagus, and airways can also make it more difficult for babies to begin and continue breastfeeding [67, 68].

Twin births and higher-order birth had an increased risk of infant mortality than single births, it was in line with study findings in the USA [69]. The possible explanation for the increased hazard of infant mortality among twins could be because twin births are more likely to experience obstructed labor, birth asphyxia, and competition for nutrition, which in turn increases the risk of respiratory infections and other related complications that can increase their risk of mortality during their first year of life [70, 71]. This also has a programmatic implication of giving special care to women with a twin pregnancy during ANC as well as intrapartum care. Regarding birth order, the birth order increases the financial burden of the family, and poverty affects infant survival through insufficient food intake, greater exposure to infections, and lack of access to vaccinations and basic health care [72].

As evidenced by previous study findings reported in Brazil [73], India [74], and developing countries [24], the birth interval was found a significant predictor of infant mortality in this study. This might be due to shorter birth interval that has been significantly correlated with maternal depletion, sibling competition, and infection transmission [75, 76]. Small size and large size babies at birth were significantly associated with excess risk of infant mortality than average size babies at birth. It was in line with studies in Pakistan [48] and Sweden [77], the possible explanation could be because low birth weight is a good indicator of the newborn's chances for survival, growth, long-term health, and psychosocial development and this could increase the risk of death in the infantile period [78, 79]. Besides, macrosomic infants are closely linked with under-lined medical conditions like DM and other lipodystrophies and are prone to birth trauma during delivery they are at risk of mortality than average size babies [80].

This study has several strengths. First, this study used weighted data to make it representative at national and regional levels, and it can be generalized to all live births in East Africa. Secondly, Gompertez gamma share frailty was fitted by considering the dependency nature of the DHS data to get reliable predictors of infant mortality. Furthermore, the study was based on a large sample size, this could increase the power of the study to get the true effect of the predictors. This finding should be interpreted in light of the following limitations. First, this study was based upon recall by mothers and it is prone to recall bias. Besides, variables such as underlined medical conditions such as congenital heart diseases, pneumonia, malaria, etc. were not included in this study since these variables were not collected in DHS.

## Conclusions

The infant mortality rate remains a major public health problem in East Africa with significant variations across the countries. The infant mortality rate is considered the most important sensitive indicator of the socioeconomic and health status of a community and its description is very vital for the evaluation and planning of public health strategies. Place of delivery, ANC visit, twin births, sex of a child, maternal education, women's autonomy, wanted pregnancy, birth size, birth interval, mode of delivery, maternal age, and birth order were found to be significant predictors of infant mortality. Therefore, enhancing health facility delivery, ANC visit, birth spacing, empowering women, and promoting maternal education should be done to reduce the incidence of infant mortality in East Africa.

## Data Availability

Data is available online and you can access it from www.measuredhs.com.

## Abbreviations

AHR: Adjusted Hazard Ratio
AIC: Akakie Information Criteria
ANC: Antenatal Care
AHR: Adjusted Hazard Ratio
CI: Confidence Interval
DHS: Demographic Health Survey
DM: Diabetic Mellitus
LLR: Log-likelihood Ratio
PH: Proportional Hazard
SDGs: Sustainable Development Goals
SSA: Sub-Saharan Africa
WHO: World Health Organization
